# Alkyd resin from rubber seed oil/linseed oil blend: A comparative study of the physiochemical properties

**DOI:** 10.1016/j.heliyon.2019.e01621

**Published:** 2019-05-07

**Authors:** G.O. Otabor, I.H. Ifijen, F.U. Mohammed, A.I. Aigbodion, E.U. Ikhuoria

**Affiliations:** a Department of Chemistry, University of Benin, Benin City, Nigeria; b Product Development Laboratory, Rubber Research Institute of Nigeria, P. M. B., 1049, Benin City, Nigeria

**Keywords:** Materials chemistry

## Abstract

This study was aimed at the synthesis and characterization of alkyd from rubber seed/linseed oil blends. Different percentages of rubber seed oil were blended with linseed oil for the preparation of alkyd via condensation polymerisation of monoglyceride with phthalic anhydride. Physiochemical properties like colour, acid value, saponification value, iodine value, drying schedule, chemical resistance were evaluated for the different alkyd samples synthesized. The colour of the different samples was unaffected by blending. The acid values of the alkyds obtained were observed to be in the range of 8.59–10.1 mgKOH/g. The iodine values of the different alkyd samples prepared increased with increase in the percentage of linseed oil in the blends. The blends showed resistance to brine, water and acid. However, only some exhibited fair resistance to alkali. The intrinsic viscosity was also determined by extrapolation from Huggins and Kraemer viscosity relationship. The results showed that the alkyd resins synthesized from the blend of linseed and rubber seed oil showed favourable properties that make linseed oil good blending oil for the synthesis of alkyd resin.

## 1 Introduction

**Scheme 1:**
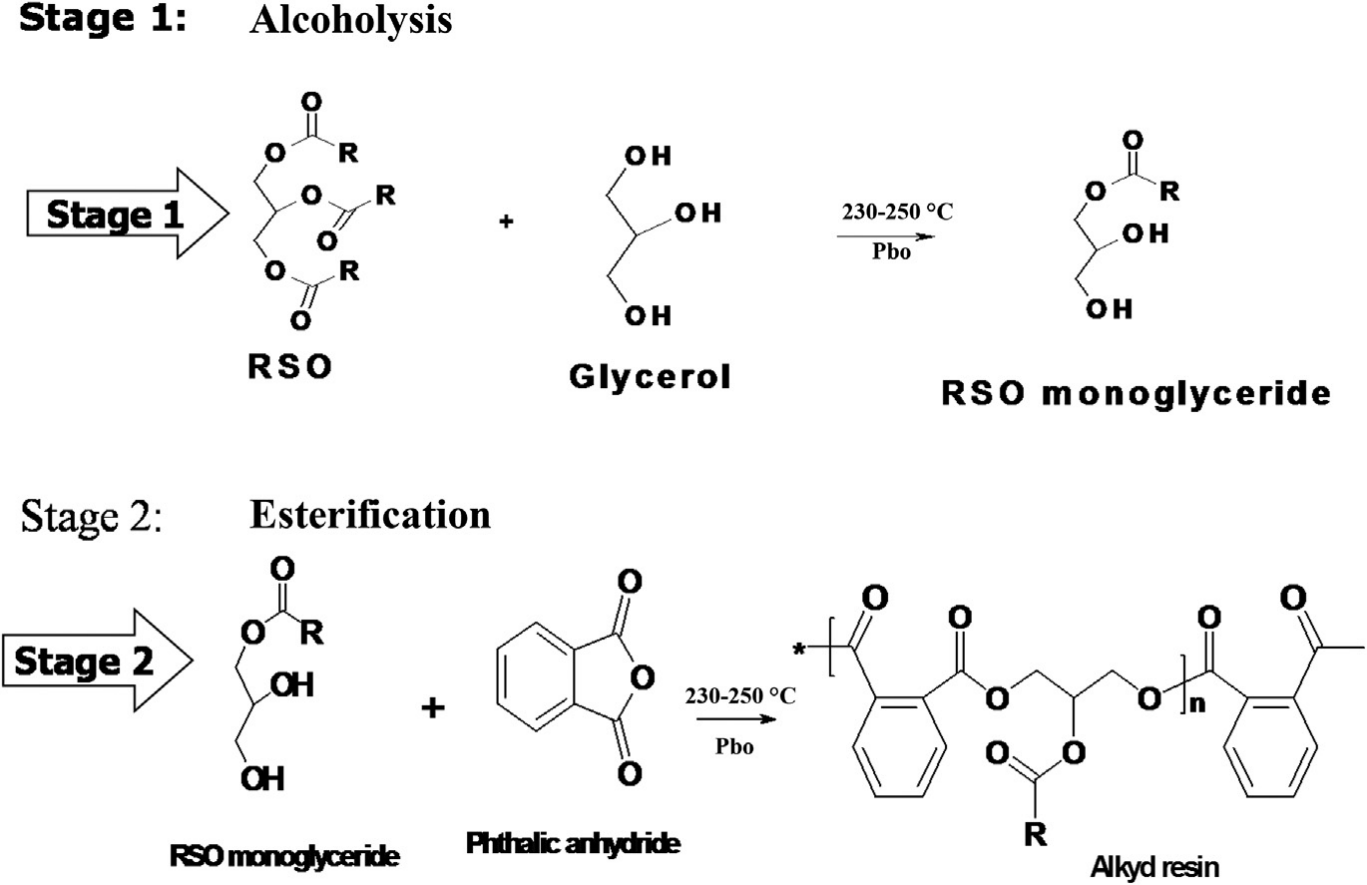
Monoglyceride process of oil-modified alkyd synthesis.

Alkyds are polyesters derived from the reaction of polyols and dicarboxylic acids or their anhydrides modified by the addition of fatty acids [1]. They can also be defined as products of a polycondensation reaction between polybasic acids and polyhydric alcohols modified with fatty acids [2]. The presence of the fatty acid confers a propensity to form plastic coating during application [1] (see Scheme 1).

Alkyd resin is one of the most indispensable raw materials in the coating and paint industries; it is the main binder accounting for a large volume of coatings and paints used for decorative purposes [3, 4]. They are inexpensive owing to the inexpensive materials from which they are produced and therefore have found applications more than other binders. In addition, they are easy to manufacture and dissolve in a cheap solvent like xylene and white spirit. Other additional qualities include ease of application under variable environmental conditions, gloss and gloss retention, durability, drying abilities, film flexibility, good adhesion, etc [5]. Alkyd resins applications are not only limited to decorative paints but are also applied in air-drying paints, machine tool finishes, inks, matt and semi-matt varnishes of wood furniture, protection of surface from chemical attack, mechanical stress and environmental effect, etc [5].

The presence of oil and glycerol as parts of alkyd resins confers the quality of them being eco-friendly compared to conventional petroleum-based polymers that constitute environmental pollution and degradation; they have attracted particular attention because of their inherent non-toxic nature and biodegradability [6, 7, 8, 9, 10].

Several types of seed oils such as linseed oil, soybean oil, castor oil and tall oil have been utilised for the syntheses of polymeric resins like alkyds depending on the nature of unsaturated fatty acid present [11, 12]. These fats and oils have the ability to slowly absorb oxygen forming dry, tough, transparent and durable films when spread and exposed to air. These drying oils contain a variety of polyunsaturated fatty acids like linoleic and linolenic acid and their triglycerides [13].

Natural rubber (*Heveabrasiliensis*) is a high utility base resource with potential for several industrial utilizations such as putty, soap, biodiesel, alkyd resins, etc. Currently, in some southern states of Nigeria, there is an abundance of natural rubber plantation that can serve as a source of rubber seed oil (RSO), a practical raw material for the production of alkyd resins on an industrial scale. It has been stated by a previous survey that about 42,980 metric tons of rubber seeds could be generated annually from a natural rubber plantation in Nigeria. However, conditions such as abnormal leaf disease, genetics, phytophora disease, weather and powdery mildew disease usually influence the quantity of rubber seed that could be produced in any given year [14].

The unique nature of rubber seed oil (a semi-drying oil) is because of its level of unsaturation, relative abundance and possession of similar properties with linseed oil (a drying oil) which is widely utilized in the synthesis of alkyd resins [15].

In Nigeria, there is a high demand for vegetable oil such as palm oil, soybean oil, coconut oil linseed oil, olive oil, etc., for nutritional, cosmetic and pharmaceutical purposes [16]. The use of rubber seed oil for non-edible purposes increases its availability and relative abundance renders it a reasonable substitute for linseed oil (*Linumusitatissimum L.*) which is costing the country a deal to import [17].

In this study, alkyd resins were synthesised via the monoglyceride process from rubber seed oil (RSO), linseed oil (LSO) and their blends. The physicochemical properties, drying performance and chemical resistance were then evaluated to investigate the effect of blending on the properties of RSO and LSO.

## 2 Materials and methods

### 2.1 Materials

The rubber (*Hevea brasiliensis*) seeds were collected from Rubber Research Institute of Nigeria, Benin City, Nigeria. The extraction of the rubber seed oil was performed using Soxhlet apparatus and *n*-hexane as extracting solvent. Linseed (*Linumusitatissimum L.*) oil was obtained commercially from a supermarket in Benin City, Nigeria. Analytical grade phthalic anhydride, glycerol, xylene, lead oxide, sodium hydroxide, sulphuric acid, potassium hydroxide, and sodium chloride (brine) were used for the preparation and characterisation of the samples. Distilled water was used throughout the experiment.

### 2.2 Preparation of alkyd samples

The synthesis of the alkyds from RSO, LSO and their blends were done according to a published procedure [18]. The alkyds were prepared using 50 % oil mixture in both the unblended and blended samples. The preparation for different oil blends and unblended oils were formulated as follows: 100 % LSO, 100 % RSO, 80 % RSO, 70 % RSO, 50 % RSO and 30 % RSO. The following formulations were used in the preparation of the alkyd samples: (see Table 1).

**Table 1:** Formulation for the preparation of alkyd resins.

Component (g)	100 % RSO	100 % LSO	80 % RSO	70% RSO	50 % RSO	30 % RSO
RSO	503.61	---	402.91	351.55	251.82	151.09
LSO	---	503.62	100.73	151.09	251.82	352.55
Glycerol	301.18	301.18	301.18	301.18	301.18	301.18
Phthalic anhydride	195.21	195.21	195.21	195.21	195.21	195.21

In the first stage, the oil (including blends) was reacted with glycerol at a temperature range of 230–250 °C in a three-necked flask fitted with mechanical stirrer using lead oxide as catalyst. This stage is complete when the mixture (monoglyceride) dissolves in anhydrous methanol in the ratio 1:3. The reaction temperature was cooled to about 180 °C.

In the second stage, phthalic anhydride and xylene (as an azeotropic solvent) were introduced into the flask. Dean and Stark (to withdraw the water of condensation) and a thermometer were also fitted to the flask. Then the reaction temperature was raised to a range of 230 and 250 °C and periodically monitored to completion, i.e., when the acid value was below 10 mgKOH/g. Nitrogen gas was continually introduced during this stage.

### 2.3 Characterization of the alkyd samples

The parameters include colour (physical observation), acid value [19], saponification value [20]and iodine value [20, 21]. The characterization of the samples was carried out based on standard procedures.

### 2.4 Determination of free fatty acid

The Free Fatty Acid (FFA) value of the rubber seed oil (RSO) and Linseed oil (LSO) (oleic acid %) was estimated by titrating phenolphthalein until the colour turns pink.

A 0.002 kg of the oil sample was weighed into a volumetric flask.

### 2.5 Determination of saponification value

2 kg of the oil sample and25 mL of 1.0 alcoholic KOH were transferred into a volumetric flask. The mixture sample was left to boil for 45 minutes in a condenser connected to the flask for complete saponification. The flask and the condenser were sufficiently cooled but not to the point of forming a gel. Thereafter, the condenser was removed and the introduction of 1 ml of phenolphthalein into the flask containing the mixture was carried out. Titration of the solution with 0.5N hydrochloric acid (HCl) was carried out until the pink colour disappeared. Determination of a blank was conducted side by side with the sample. The calculation of the saponification value was done using the formula below:

$$\text{Iodine}\;\text{value}=\frac{56.1\times N\times \left(V2-V1\right)}{W}$$

Where, W = weight of the sample, (g)
V1 = volume of HCl used in the test, (mL)
V2 = volume of HCl used in the blank, (mL)
N = normality of HCl.

### 2.6 Determination of iodine value

In a typical determination, oil sample (1 g) was transferred into a 500 mL volumetric flask. Carbon tetrachloride (15 mL) was added to the sample with stirring. Wijs solution (25 mL) was thereafter added to the flask containing the sample with the aid of a pipette. The sample was allowed to stand in the dark for 30 minutes at room temperature. 20 mL of 10 % potassium iodide (KI) solution and 150 mL of distilled water were introduced into the flask. 0.1 N thiosulphate (Na_2_ S_2_ O_3_) solution was titrated with the mixture until the yellow colour disappeared almost completely. 1.5 mL of starch indicator solution was again introduced and the titration was continued until the disappearance of the blue colour. Blank determination was conducted simultaneously. The formula below was used to calculate the iodine value:

$$\text{Iodine}\;\text{value}=\frac{12.69\times \left(V2-V1\right)\times N}{W}$$

Where = weight of the sample, (g)
V1 = volume of HCl used in the test, (mL)
V2 = volume of HCl used in the blank, (mL)
N = normality of HCl.

### 2.7 Preparation of alkyd coatings for performance characteristics

The alkyd samples were thinned to 50 % with xylene and applied on thin glass plates. Thereafter, loosening, detachment, wrinkling or any form of distortion on the film was examined. The drying schedule of the samples in terms of the time of set-to-touch, tack-free and dry-through was recorded at room temperature.

The chemical resistance of the dried films to different solvent media by immersion method for a period of 24 hours was then examined [22]. The composition of the service media used was made up of distilled water, brine (5 % w/v), 0.1M H_2_SO_4_ and 0.1 M KOH.

### 2.8 Viscosity measurement

Viscosity measurements were carried out with a solution of alkyd in toluene using Ubbelohde viscometer as described by a published procedure [23]. Three additional dilutions were made in the viscometer, allowing efflux times to be measured at a concentration of 2.5, 2.0, 1.5 and 0.5 g/100 cm^3^. Viscosity measurement was carried out at a temperature of 30 ± 0.5 °C. Intrinsic viscosities were determined by extrapolation of the plots of η_sp_/C against C where C is the concentration (g/100 ml) and η_sp_ is the specific viscosity. The values of the Huggin's constant K were calculated using the Huggins and Kraemer viscosity relationship shown in Eq. (1).

(1) $${\eta_{sp}/C=\left[\eta \right]+K\left[\eta \right]}^{2}C$$

## 3 Results and discussion

### 3.1 Physicochemical properties of the oils

The physicochemical properties of the rubber seed oil and linseed oil are given in Table 2.

**Table 2:** Physiochemical properties of the RSO and LSO.

Properties	RSO	LSO
Colour	Brown	Light yellow
Acid value (mgKOH/g)	18.19	1.00
Free fatty acid (% as oleic acid)	9.648	0.503
Saponification value (mgKOH/g)	180.40	194.00
Iodine value (gI_2_/100 g)	142.20	188.00

The colours of the rubber seed oil and linseed oil used for this experiment were observed to be brown and light yellow respectively. This is important for comparison with alkyd samples prepared from the oil and subsequently in their coating application. The acid value of rubber seed oil is significantly higher than that of linseed oil arising from possible deterioration of the rubber seed oil. Linseed oil has a higher saponification value than rubber seed oil suggesting a higher average molecular weight. Linseed oil shows a considerably higher level of unsaturation than rubber seed oil indicating a higher drying property than rubber seed oil. The observed iodine values are188.00 and 142.20 gI_2_/100 g respectively.

### 3.2 Physiochemical properties of the alkyd samples

Table 3 shows the physicochemical properties of the prepared alkyd samples. The result shows that the colour of the alkyds from the unblended oils is dark brown and are unaffected by the blending. The colour could be attributed to the high temperature condition of the synthesis process (see Scheme 1).

**Table 3:** Physiochemical properties of the alkyd samples.

Properties		Colour	Acid value (mgKOH/g)	Saponification value (mg/KOH/g)	Iodine value (gI_2_/100 g)
100%RSO		Dark brown	9.75	212.45	43.73
100% LSO		Dark brown	8.92	252.35	79.25
80% RSO		Dark brown	10.1	222.43	66.09
70% RSO		Dark brown	9.84	229.16	68.76
50% RSO		Dark brown	9.55	234.72	75.33
30% RSO		Dark brown	8.57	240.35	77.78

The obtained acid values of the synthesized alkyd resin samples were observed to be in the range of 8.57–10.1 mgKOH/g. The results show that acid values decreases with an increased percentage of linseed oil. This could be as a result of the very low acid value of the linseed oil shown in Table 2.

The saponification value of the alkyd sample prepared from 100 % LSO is observed to be higher than that of 100 % RSO. The saponification values of the blends (RSO/LSO) generally increase with a decrease in the percentage of linseed oil and were observed to be higher than that of 100% RSO.

The high iodine value recorded by100 % LSO when compared to the 100 % RSO may be ascribed to the drying property of LSO. Consequently, the iodine values of blended samples show a marked increase with an increase in the percentage of LSO.

### 3.3 Drying property of the alkyd films

The drying schedule of the different alkyd samples was carried out in terms of set-to-touch, tack-free and dry-through (see Table 4). These parameters are important in understanding the rate and extent of drying of the coating when applied to a surface.

**Table 4:** Drying property of the alkyd film.

Drying property	100 % LSO	100 % RSO	80 % RSO	70 % RSO	50 % RSO	30 % RSO
Set to touch (min)	6	48	42	36	24	12
Tack free (min)	24	132	108	84	60	36
Dry-through (min)	144	288	222	204	180	168

The set-to-touch time of 100 % LSO is significantly higher than that of 100 % RSO; this is an indication that LSO has a faster drying property than RSO. In comparison with 100 % RSO, the blends showed shorter set-to-touch time with an increased percentage of LSO. The set-to-touch times for the blends were found to be in the range of 12 mins for 30 % RSO and 42 mins for 80 % RSO. This trend was also observed for the iodine values in Table 3, i.e., the set-to-touch time is an inverse function of the iodine value.

The tack-free and dry-through time also followed the same pattern as the set-to-touch time. The tack-free and dry-through times for the blends were observed to be about 36–108 mins and 168–222 mins respectively.

### 3.4 Chemical resistance of the alkyd film to different media

The chemical resistance of the alkyds in different media is presented in Table 5. All alkyds prepared generally showed resistance to brine, water and acid. However, some of the films prepared by the synthesized alkyd samples were removed by alkali. This is due to the susceptibility of the ester linkage to alkaline hydrolysis.

**Table 5:** Chemical resistance of the alkyd film to different media.

Solvents	100 % RSO	100 % LSO	80 % RSO	70 % RSO	50 % RSO	30 % RSO
Distilled water	2	2	2	2	2	2
NaCl (5 %w/v)	1	1	1	1	1	1
H_2_SO_4_ (0.1 M)	2	2	2	2	2	2
KOH (0.1 M)	4	3	3	3	4	4

NoEffect = 1, Whitening = 2, Shrinkage of film = 3, Removal of film = 4.

### 3.5 Estimated solution parameters of alkyd samples

Table 6 shows the values of the intrinsic viscosity and their corresponding Huggin's constant for the unblended and blended alkyd samples prepared. High Huggin's constant implies that the entire synthesized alkyd samples are highly viscous and less soluble in the solvent medium. The viscosity was observed to increase as the percentage of rubber seed oil used during the alkyd synthesis increased. 100 % RSO and 100 % LSO alkyds were observed to possess the highest viscosity, followed by 80 % RSO alkyd and so on. The observed pattern in Table 6 can be attributed to the more viscous nature of rubber seed oil compared to linseed oil.

**Table 6:** Estimated solution parameters of alkyd samples.

Alkyd samples	Intrinsic viscosity [η](cm^3^/g)	Huggin's constant (K_H_)
100 % RSO	0.0469	0.546
100 % LSO	0.0442	4.914
80 % RSO	0.0441	0.926
70 % RSO	0.0359	2.040
50 % RSO	0.0313	2.450
30 % RSO	0.0320	3.906

## 4 Conclusions

The synthesis and characterisation of alkyd resin blends from rubber seed oil (RSO) and linseed oil (LSO) using 50% oil mixture have been successfully carried out. The blends exhibited good resistance to brine, water and acid. However, increased linseed oil in the blends resulted in fairly lower viscosities compared to the unblended alkyd samples prepared. Experimental results showed that LSO has favourable properties that make it good blending oil in enhancing the drying properties of RSO. The Blending of RSO with LSO should be encouraged in order to improve the drying property of RSO for the synthesis of an alkyd with good properties.

## Declarations

### Author contribution statement

Ikhazuagbe Ifijen, Farouk Mohammed: Analyzed and interpreted the data.

G.O. Otabor: Performed the experiments; Analyzed and interpreted the data.

A. Aigbodion: Analyzed and interpreted the data; Contributed reagents, materials, analysis tools or data.

E. Ikhuoria: Conceived and designed the experiments; Contributed reagents, materials, analysis tools or data; Wrote the paper.

### Funding statement

This research did not receive any specific grant from funding agencies in the public, commercial, or not-for-profit sectors.

### Competing interest statement

The authors declare no conflict of interest.

### Additional information

No additional information is available for this paper.
